# The use of clade‐specific PCR assays to identify novel nitrilase genes from environmental isolates

**DOI:** 10.1002/mbo3.700

**Published:** 2018-12-30

**Authors:** Tríona‐Marie Dooley‐Cullinane, Catherine O'Reilly, Bilal Aslam, David P. Weiner, David O'Neill, Erica Owens, Denise O'Meara, Lee Coffey

**Affiliations:** ^1^ Department of Science Waterford Institute of Technology Waterford Ireland; ^2^ Molecular Biotechnology and Biocatalysis Group Pharmaceutical and Molecular Biotechnology Research Center Waterford Institute of Technology Waterford Ireland; ^3^ BASF Enzymes LLC San Diego California

**Keywords:** clade specific, degenerate primers, environmental isolates, gene detection, nitrilase, touchdown PCR

## Abstract

Nitrilase enzymes (EC 3.5.5.1) are responsible for the direct hydration of nitriles to their corresponding carboxylic acids and ammonia. The utilization of nitrilase enzymes in biocatalysis toward bio‐pharmaceuticals and industrial applications facilitates the move towards green chemistry. The body of research presented describes a novel clade‐specific touchdown PCR protocol for the detection of novel nitrilase genes. The presented study identified partial sequences of 15 novel nitrilase genes across 7 genera, with partial DNA sequence homology (%) displayed across an additional 16 genera. This research will prove valuable in the screening of microorganisms for the identification of novel clade‐specific nitrilase genes, with predicted enantioselective profiles as determined by their clade characterizations.

## 1 INTRODUCTION

Nitrilase enzymes (EC 3.5.5.1) are involved in the direct conversion of nitriles to their corresponding carboxylic acids and ammonia through a one‐step reaction (Banerjee et al., 2002). Microbial nitrilases are abundant in nature due to their participation in degradation pathways for both natural and xenobiotic nitriles (Baxter & Cummings, 2006; Coffey et al., 2009; Prasad & Bhalla, 2010).

Members of the nitrilase superfamily consist of a catalytic triad of amino acids; glutamic acid, lysine, and cysteine. The nitrilase branch can easily be distinguished within the superfamily by a conserved cysteine‐tryptophan‐glutamic acid arrangement, encompassing the cysteine residue of the catalytic triad. Studies have noted a complete loss of nitrilase activity upon mutation of the cysteine residue (Howden & Preston, 2009; Kobayashi et al., 1993), moreover, the sulfhydryl group associated with nitrilases and their activity accounts for their classification as thiol enzymes, as discussed by O'Reilly and Turner (2003).

The biotechnological potential and capabilities of nitrilases have been unmistakable, facilitating the move toward green chemistry within both the pharmaceutical and biotechnological industries (Gong et al., 2012; Martínková & Kren, 2010; Martínková et al., 2009; Sandhya et al., 2005; Singn et al., 2006; Solano et al., 2012; Robertson et al., 2004; Winkler et al., 2009). Environmental samples are continuously exploited for the isolation and identification of novel nitrilase enzymes, with the first plant and bacterial nitrilases identified in 1958 and 1964, respectively, (Robinson & Hook, 1964; Thimann & Mahadevan, 1958).

As the body of published work on nitrilases increases, the sequence diversity between the nitrilase genes becomes more evident. Following the construction and functional screening of numerous metagenomic libraries, Robertson et al. (2004) classified nitrilase genes into various clades based on sequence homology. Over 137 unique nitrilases were discovered and classified into several clades with over 40%–60% amino acid sequence similarities observed between each clade. Remarkably, variation in nitrilase sequence can be observed within the clades where amino acid sequence similarities can be as low as 75% (Robertson et al., 2004).

Due to these sequence variations, many techniques are employed in novel enzyme discovery, such as metagenomic library construction and functional screening (Robertson et al., 2004; Soares Bragance et al., 2017) whole genome sequencing, and data mining (Kaplan et al., 2011; Sharma & Bhalla, 2012; Veselá et al., 2016).

Previous research by our research group has focused on the isolation and screening for nitrile metabolising isolates (Coffey et al., 2009, 2010), designing screening protocols for the aldoxime dehydratase gene (*oxd*) in the aldoxime–nitrile metabolizing pathway (Dooley‐Cullinane et al., 2016) and the use of applied metagenomics for the discovery of novel nitrile metabolising enzymes (Soares Bragança et al., 2017). To the best of our knowledge, there has been no method published to date which allows for the detection of clade‐specific nitrilase genes across numerous genera. The work presented here allows for the detection of clade‐specific nitrilase genes across multiple genera via touchdown PCR, with 15 novel partial nitrilase sequences presented across 7 genera.

## 2 MATERIALS AND METHOD

### 2.1 Soil sources

Soil samples were collected from various locations across Ireland as detailed by (Coffey et al., 2009).

### 2.2 Primer design

Primer sets were designed from a ClustalW alignment (Thompson et al., 1994) of clade‐specific nitrilase genes as published by (Robertson et al., 2004) and all unclassified‐clade nitrilase sequences available in the Genbank database (Benson et al., 2013), including putative sequences. Each primer pair was designed with a maximum degeneracy of 192 (Table S1). All primers designed and used in this study were supplied by Eurofins, MWG operon, Germany.

### 2.3 Nitrilase gene‐screening approach—Touch‐down PCR protocol

Each 15 μl PCR mixture contained 7.5 μl Gotaq^™^ Green Master Mix (Promega, UK), 15 pmol of each primer and (a) environmental bacterial cells suspensions adjusted to a final O.D._600_ = 0.04, or (b) 15 ng of metagenomic fosmid clone DNA. The following PCR conditions were used: 1 cycle of 95°C for 5 min, 2 cycles of 95°C for 1 min, 58°C for 1 min, 72°C for 40 s, followed by a 1°C reduction in annealing temperature every 2 cycles to 51°C inclusive; followed by 20 cycles of 95°C for 1 min, 50°C for 1 min and 72°C for 40 s. A final extension stage of 8 min was utilized. Negative controls contained water in place of cells/DNA, with positive controls consisting of nitrilase DNA, supplied by Verenium Corporation (now part of BASF), listed in Table S2.

### 2.4 Cloning of potential nitrilase amplicons

All potential nitrilase PCR products were gel extracted, using the Qiagen gel extraction kit (Qiagen cat. no. 28704) as per manufacturer's instructions, and cloned into the pDrive vector (Qiagen, cat. no. 231122). Plasmids were transformed into Novablue Gigasingles^™^ (Novagen, cat. no. 71227), positive clones were selected via blue/white screening.

### 2.5 Sequence analysis

Sequencing of each potentially positive nitrilase plasmid insert was carried out in triplicate using the vector‐specific primers M13 Forward (‐20) and M13 reverse, with all sequencing carried out by GATC Biotech, Germany. Nucleotide sequences were analyzed using Blastn software from the NCBI database (Altschul et al., 1990). Phylogenetic trees were constructed using Dendroscope (Uson & Cornavacca, 2012) with a clustalW alignment of the sequenced nitrilase region with gaps allowed, bootstrap trials at 1,000 and bootstrap labels on nodes.

## 3 RESULTS AND DISCUSSION

A subset of 80 isolates from a larger microbial pool isolated previously (Coffey et al., 2009) were screened via the described touchdown PCR method, yielding fifteen potential nitrilase PCR products with positive identification of partial nitrilase sequence confirmed upon sequence analysis (Table S1). The isolates which partial nitrilase sequence were derived from were identified by 16S rRNA gene PCR amplification and sequencing as per with the 15 novel partial nitrilase sequences presented in ST3.

**Figure 1:**
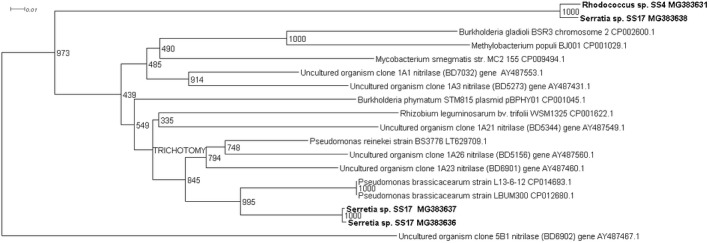
Positive control uncultured clones 1A1 (BD7032), 1A3 (BD5273), 1A21 (BD5344), 1A23 (BD6901) and, 1A23 (BD5156) were used in the contruction of the 1A phylogenetic tree. The uncultured organism clone 5B1 (BD6902) gene was use to root the phylogenetic tree

**Figure 2:**
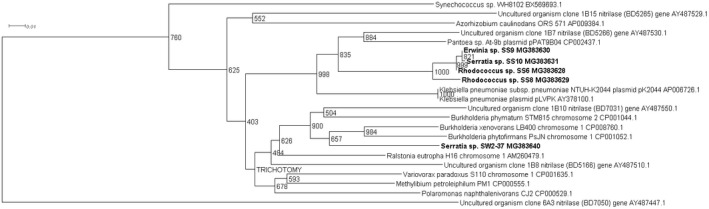
Positive control uncultured clones 1B15 (BD5265), 1B7 (BD5266) and, 1B10 (BD7031) were used in the contruction of the 1B phylogenetic tree.The uncultured organism clone 6A3 (BD7050) gene was use to root the phylogenetic tree

**Figure 3:**
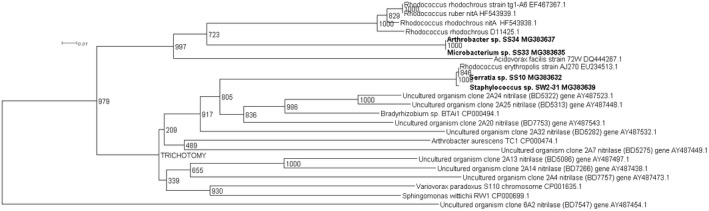
Positive control uncultured clones 2A24 (BD5332), 2A25 (BD5313), 2A20 (BD7753), 2A32 (BD5282), 2A7 (BD5275), 2A13 (BD5086) and, 2A14 (BD7757) were used in the contruction of the 2A phylogenetic tree.The uncultured organism clone 6A2 (BD7547) gene was use to root the phylogenetic tree

**Figure 4:**
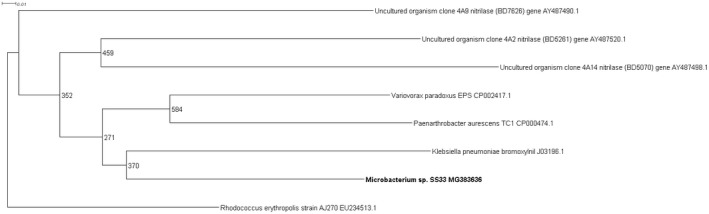
Positive control uncultured clones 4A8 (BD7626), 4A2 (BD5261) and, 4A14 (BD5070) were used in the contruction of the 4A phylogenetic tree.The nitrilase from *Rhodococcus erythropolis* AJ270 (EU234513.1) gene was use to root the phylogenetic tree

**Figure 5:**
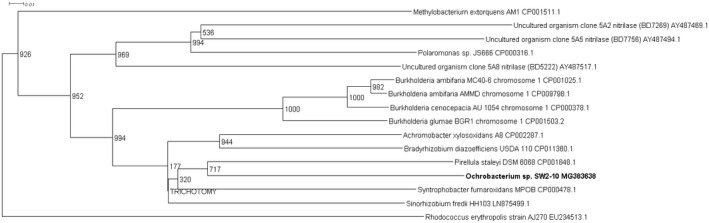
Positive control uncultured clones 5A2 (BD7269), 5A5 (BD7756) and, 5A8 (BD5222) were used in the contruction of the 5A phylogenetic tree. The nitrilase from *Rhodococcus erythropolis* AJ270 (EU234513.1) gene was use to root the phylogenetic tree

Four novel partial nitrilase sequences were discovered in the 1A clade with sequence homologies to a number of nitrilase genes across multiple genera, Figure 1. The highest number of partial nitrilase sequences were identified in the 1B clade, with five sequences discovered across *Rhodococcus* sp., *Erwinia* sp., and *Serratia* sp., as seen in Figure 2. Four novel partial nitrilase sequences were identified within the 2A clade encompassing *Microbacterium* sp., *Arthrobacter* sp., *Serratia* sp., and *Staphylococcus* sp. with sequence homology observed across a number of nitrilases from multiple genera, specifically *Rhodococcus* sp. as seen in Figure 3. One partial novel nitrilase sequence was identified in the 4A clade in *Microbacterium* sp. SS33, with an observed sequence homology to a low number of partial nitrilase sequences available on the database, Figure 4. One novel 5A nitrilase was discovered from *Ochrobactrum* sp. with a high sequence homology to a number of 5A *Burkholderia* sp. nitrilase sequences, Figure 5. Enzymes or whole cell biotransformations are heavily utilized to produce biotechnological and pharmaceutical intermediates. The microbial nitrile metabolizing pathway is of particular interest, with nitrilase enzymes affording the ability to produce enantiopure carboxylic acids (Coady et al., 2013; Martínková et al., 2017). According to Fleming et al., (2010), there were over 30‐nitrile containing pharmaceuticals on the market and another 11 nitrile‐containing drugs were approved since 2011 (Dooley‐Cullinane et al., 2016). One well‐known example of chemoenzymatic synthesis relates to the API Atorvastatin, for the cholesterol lowering drug Lipitor^®^ (Solano et al., 2012). It is estimated that, by 2030, the “products of white biotechnology and bioenergy will account for 30% of industrial production worth €300 billion” (European Union, 2007). The drive toward a ‘green chemistry’ approach is increasing the demand for the discovery of novel genes and enzymes (Rasor & Voss, 2001). As the body of research increases, the true extent to which these enzymes can be utilized becomes more evident.

Previous research published by Robertson et al. (2004) classified newly identified nitrilase gene sequences into clades based on sequence homology. Prior to the publication, the number of nitrilase sequences on the database was limited, with much sequence bias. The research identified 137 novel nitrilase gene sequences which when presented in an unrooted tree supported several distinct clades (Robertson et al., 2004). Interestingly, some of the genes discovered within each clade revealed similar substrate specificities. Within the biotechnological and pharmaceutical industry, significant focus is placed upon the purity of the expected products and the enantiomer which is produced, further showing the importance of gene sequences and their relationship to the predicted enzyme activity (Coady, 2014; Martínková & Kren, 2010; Rasor & Voss, 2001; Solano et al., 2012).

During primer design, it was paramount that attention was given to the level and location of degeneracy afforded, to ensure that the detected gene would not lend to sequence bias. Confirmed nitrilase gene sequences from wild‐type microorganisms and cloned recombinant enzymes available on the NCBI database were utilized during primer design. For example, the 1A clade primers were designed based on the alignment of 36 nitrilase sequences from the database combined with all 1A clade uncultured clone nitrilase gene sequences presented by Robertson et al. (2004).

The same approach was applied during the primer design for each clade, with differences observed in the number of sequences exploited and the degree of degeneracy. Sequence homologies between the exploited nitrilase genes permitted the identification of conserved regions within the genes, with the degeneracy accounting for the variation evident within each clade.

Four 1A clade partial nitrilases sequences were identified, as seen in the phylogram presented in Figure 1. Two of these partial genes, identified in isolate *Rhodococcus* sp. SS4 (MG383627) and *Serratia* sp. SS17 (MG383638) displayed high sequence homology, despite being isolated from different soil sources and on different nitriles, acetonitrile, and benzonitrile, respectively. They display the highest sequence similarity to the uncultured gene 1A1 (AY 487553.1). The remaining two nitrilases discovered in *Serratia* sp. SS17 (MG383633 and MG383634) displayed the highest sequence homology to uncultured nitrilase 1A23 (AY487460.1) as discovered by Robertson et al. (2004), where the majority of enzymes clustered in the 1A clade displayed S‐enantioselectivity. Though the above partial nitrilase sequences were isolated on achiral substrates, clade categorization could suggest that the cells or nitrilase enzyme may permit enantioselective hydrolysis of a suitable substrate.

Five newly discovered partial nitrilase sequences were elucidated across the 1B clade. Four partial nitrilase sequences were identified in microorganisms, which were isolated from selective enrichments with soil samples and one from a microorganism, *Serratia* sp. SW2‐37 (MG383640), which was isolated from a selective enrichment of a *Ceramium codicola* sample. The constructed phylogram can be seen in Figure 2. These partial sequences coding the nitrilase genes detected in isolate *Erwinia* sp. SS9 (MG383630) and *Serratia* sp. SS10 (MG383631) displayed the highest homology despite being isolated from different soil sources on alternative nitriles during the enrichments, acetonitrile and adiponitrile, respectively, similar to isolate *Rhodococcus* sp. SS8 (MG383629) and *Rhodococcus* sp. SS6 (MG383628) The aforementioned nitrilase displayed the highest sequence homology to the uncultured clone 1B10 (AY 487550.1) (Robertson et al., 2004). The majority of the 1B clade, as conferred by Robertson et al. (2004), display S‐enantioselectivity. This also applies to the uncultured clone 1B8 (AY 487510.1), which displays the highest sequence homology to the partial identified nitrilase sequence from *Serratia* sp. SW2‐37 (MG383640).

The 2A clade, as discussed by Robertson et al. (2004) was largely R‐selective. The four‐elucidated partial nitrilase sequences from this current study displayed high sequence homology to each other but also to a number of *Rhodococcus* sp. nitrilase genes. Three of the four partial nitrilases, *Serratia* sp. SS10 (MG383632), *Microbacterium* sp. SS33 (MG383635) and *Arthrobacter* sp. SS34 (MG383637) were isolated from selective enrichments with soil, in contrast, the *Staphylococcus* sp. SW2‐31 (MG383639) partial nitrilase sequence isolated from a selective enrichment of *Palmaria palmata* samples. Interestingly, the phylogram for the 2A clade, presented in Figure 3, displays the highest number *Rhodococcus* sp. nitrilase genes. This may be due to sequence bias in previous methods used to discover these nitrilase genes or it may indicate that *Rhodococcus* sp. could in fact harbour a higher ratio of R‐selective versus S‐selective enzymes, which would be indicated by their clade categorization. From another perspective, it could signify that there is a bias in the culture conditions from environmental sources which favour *Rhodococcus* sp. or, that they account for a high percentage of the culturable portion the soil samples utilized in studies.

One partial sequence for a 4A novel nitrilase gene was detected in *Microbacterium* sp. SS34 (MG383636), displaying the highest sequence similarity to *Klebsiella pneumoniase* bromoxynil as presented in the phylogram in Figure 4. Published research by Robertson et al. (2004) accounted for the detection of 17 novel 4A nitrilases. The comparatively low number of partial nitrilase genes detected in this study is not unexpected as a section of cultured environmental isolates were screened, in comparison to the screening of a metagenomic library. It may be a preliminary indication that the nitrilase genes associated with the 4A clade are not commonly found in the culturable portion of environmental isolates samples utilized in this study. The available literature is suggestive that the majority of enzyme which are categorized into the 4A clade could be S‐selective (Robertson et al., 2004).

One partial sequence for a 5A novel nitrilase was identified from seaweed isolate *Ochrobacterium* sp. SW2‐10 (MG383638), refer to phylogenetic tree in Figure 5. It displayed the highest sequence homology to *Piruella staleyi* DSM6068 (CP 001848.1) and, based on the findings published by Robertson et al. (2004), it could hypothetically be an (R)‐selective enzyme. The phylogram displays a cluster of *Burkholderia* sp. which may indicate that these predicted R‐selective 5A clade nitrilase genes are common amongst this genera, perhaps obtained by horizontal gene transfer, such as the published findings by (Coffey et al., 2009). It is not surprising that the ratio of 5A partial nitrilase genes to either the 1A, 1B, or 2A clades was lower. Based on the published findings by Robertson et al. (2004) only nine 5A clade nitrilase were discovered from the metagenomic library, indicating that they may in fact not be common in culturable microorganisms.

For the clades 3A, 5B, and 6A, no phylogenetic analysis is available as no partial sequences for nitrilase enzymes were identified in the subset of screened isolates. This is not surprising as only seven novel nitrilases were identified by Robertson et al. (2004) across the 3A and 6A clades, with 3 and 4 clones, respectively. While there were 21 clones identified by Robertson et al., within the 5B clade, our lack of detection of a 5B clade nitrilase within our subset of isolates may be due to the fact they originate from the nonculturable portion of the environmental sample or, it may be a case that they were simply not present in our samples. It is also worth noting that the seven aforementioned nitrilase genes were identified from the construction of a metagenomic library. In contrast, the approach used in this study relied on screening wild‐type cultured isolates, which only represented a fraction of the culturable portion of the selected environmental samples. The designed primers and nitrilase screening approach did yield positive amplification of the nitrilase gene from all the positive controls associated with the 3A, 5B, and 6A clades utilized in the study, inferring that the lack of detection is due to sample selection rather than the primer and method design.

The novel method described in this study will enable researchers to prioritize samples for further analysis such as full gene sequencing via primer‐walking or bridge PCR, or whole genome sequencing on next generation sequencing platforms.

The touchdown PCR method described allows for the screening of clade‐specific novel nitrilase genes. The development of such a protocol allows for the targeted identification of enzymes with a predicted activity based on clade characterization, as reported by Robertson et al., 2004. Nitrilases from metagenomic sources or from wild‐type organisms displaying interesting metabolizing abilities can be detected, thus omitting the need for whole genome sequencing or for fosmid insert sequencing. Various methods can be employed to obtain the full nitrilase gene sequence once identified via this assay, such as primer‐walking, inverse/bridge PCR, etc. Indeed, if whole genome sequencing is feasible, at the very least this assay enables the prioritization of sample sequencing and prevalidates the elucidation of complete nitrilase sequence discovery. This method will prove beneficial to researchers focusing on the identification of novel Nitrilase enzymes with a specific target activity or enantioselective product synthesis for applications in biotechnology or the biocatalytic/bio‐pharmaceutical sector.

In conclusion, the work describes the development of a touchdown PCR method for the detection of novel nitrilase genes from environmental isolates. These newly designed clade‐specific PCR assays will allow for the efficient search for nitrilase genes based on clade‐classifications enabling the identification and elucidation of novel nitrilase genes with a predicted enantioselectivity profile. The method will prove to be valuable to the ongoing search for novel nitrile metabolising enzymes toward industrial and biopharmaceutical applications.

## CONFLICT OF INTEREST

No conflict of interest to declare.

## SIGNIFICANCE AND IMPACT OF STUDY

The identification for novel enzymes/biocatalysts toward the production of enantiopure pharmaceutical intermediates, fine chemicals and, chiral acids is a challenge which the biopharmaceutical and fine chemical industries are facing. These newly designed suites of clade‐specific PCR assays will allow for the efficient search for nitrilase genes based on clade classifications enabling the identification and elucidation of novel nitrilase genes with a predicted enantioselectivity profile. The method will prove to be valuable to the ongoing search for novel nitrile metabolizing enzymes toward industrial and biopharmaceutical applications.

## Data Availability

Sequencing data is available through the National Centre for Biotechnology Information (https://www.ncbi.nlm.nih.gov) using the corresponding accession numbers provided throughout.
