# Supplementary material for: The use of clade‐specific PCR assays to identify novel nitrilase genes from environmental isolates
Source: Microbiologyopen. 2018 Dec 30;8(4):e00700. doi: 10.1002/mbo3.700 (PMC6460282; doi:10.1002/mbo3.700)
Supplement: Supplementary file 1 [file MBO3-8-e00700-s001.docx]

Table S1 Clade-specific primer pairs with sequences (5’-3’) and expected amplicon size (bp)

| **Nitrilase Clade** | **Forward Primer** | **Reverse Primer** | **Expected Amplicon size (bp)** |
| --- | --- | --- | --- |
| 1A | atgrtctggggvcargghga | tcdccytgbccccagaycat | 287 |
| 1B | caygarcgsatgrtstgggg | tccatcatbckyttkcgytt | 440 |
| 2A1 | gsvytbtgctgytgggarca | rtartgvccrgcvggrtc | 487 |
| 2A2 | gsvytbtgctgytgggarca | gartartgsccgrcsggrtc | 512 |
| 3A | caycgcaarctscarccsac | ttcatsakbscstcratctg | 125 |
| 4A | caycgcaarytgrwgccsac | catcywrtkytcccagca | 125 |
| 5A | tgctgggarmayyayatgcc | tcsgsvcgcgmrtartgscc | 412 |
| 5B | tgctgggarmayyayatgcc | tcrccdwyrttratccaytc | 250 |
| 6A | caycgyaagctcrtgccvac | catdhrgttytcccagcaga | 250 |

*Universal code for degenerate bases R = A/G, Y = C/T, M = A/C, K = G/T, S = C/G, W = A/T, B = C/G/T, D = A/G/T, H = A/C/T, V = A/C/G, and N = A/C/G/T.

Table S2 Clade-specific positive controls utilized during the touchdown PCR screening with corresponding accession numbers.

| **Clade** | **Clone I.D.** | **Accession Number** | **Clade** | **Clone I.D.** | **Accession Number** |
| --- | --- | --- | --- | --- | --- |
| 1A | 1 | [**AY487553.1**](https://www.ncbi.nlm.nih.gov/nucleotide/40890072?report=genbank&log$=nuclalign&blast_rank=1&RID=85A2X886015) | 2A | 24 | [**AY487523.1**](https://www.ncbi.nlm.nih.gov/nucleotide/40890250?report=genbank&log$=nuclalign&blast_rank=1&RID=8BKUHVYT014) |
| 1A | 3 | [**AY487431.1**](https://www.ncbi.nlm.nih.gov/nucleotide/40890312?report=genbank&log$=nuclalign&blast_rank=1&RID=85A6GF71015) | 2A | 25 | [**AY487448.1**](https://www.ncbi.nlm.nih.gov/nucleotide/40890268?report=genbank&log$=nuclalign&blast_rank=1&RID=85A0NW6V014) |
| 1A | 21 | [**AY487549.1**](https://www.ncbi.nlm.nih.gov/nucleotide/40890174?report=genbank&log$=nuclalign&blast_rank=1&RID=8BKGXRNU014) | 3A | 2 | [**AY487516.1**](https://www.ncbi.nlm.nih.gov/nucleotide/40890236?report=genbank&log$=nuclalign&blast_rank=1&RID=859KYX7X014) |
| 1A | 23 | [**AY487460.1**](https://www.ncbi.nlm.nih.gov/nucleotide/40890174?report=genbank&log$=nuclalign&blast_rank=1&RID=8BKGXRNU014) | 4A | 2 | [**AY487520.1**](https://www.ncbi.nlm.nih.gov/nucleotide/40890244?report=genbank&log$=nuclalign&blast_rank=1&RID=859NYTJ2015) |
| 1A | 26 | [**AY487560.1**](https://www.ncbi.nlm.nih.gov/nucleotide/40890174?report=genbank&log$=nuclalign&blast_rank=1&RID=8BKGXRNU014) | 4A | 9 | [**AY487490.1**](https://www.ncbi.nlm.nih.gov/nucleotide/40890184?report=genbank&log$=nuclalign&blast_rank=1&RID=8BKP55WP015) |
| 1B | 7 | [**AY487530.1**](https://www.ncbi.nlm.nih.gov/nucleotide/40890224?report=genbank&log$=nuclalign&blast_rank=1&RID=8598X671014) | 4A | 14 | [**AY487498.1**](https://www.ncbi.nlm.nih.gov/nucleotide/40890200?report=genbank&log$=nuclalign&blast_rank=1&RID=859CZ757014) |
| 1B | 8 | [**AY487510.1**](https://www.ncbi.nlm.nih.gov/nucleotide/40890224?report=genbank&log$=nuclalign&blast_rank=1&RID=8598X671014) | 5A | 2 | [**AY487469.1**](https://www.ncbi.nlm.nih.gov/nucleotide/40890142?report=genbank&log$=nuclalign&blast_rank=1&RID=8BKECND3014) |
| 1B | 10 | [**AY487**](https://www.ncbi.nlm.nih.gov/nucleotide/40890224?report=genbank&log$=nuclalign&blast_rank=1&RID=8598X671014)**550.1** | 5A | 5 | [**AY487494.1**](https://www.ncbi.nlm.nih.gov/nucleotide/40890192?report=genbank&log$=nuclalign&blast_rank=1&RID=8BKSURS1014) |
| 1B | 15 | [**AY487529.1**](https://www.ncbi.nlm.nih.gov/nucleotide/40890262?report=genbank&log$=nuclalign&blast_rank=1&RID=859X98DP015) | 5A | 8 | [**AY487517.1**](https://www.ncbi.nlm.nih.gov/nucleotide/40890238?report=genbank&log$=nuclalign&blast_rank=1&RID=859GX4K9014) |
| 2A | 4 | [**AY487**](https://www.ncbi.nlm.nih.gov/nucleotide/40890102?report=genbank&log$=nuclalign&blast_rank=1&RID=8BK6ZA0U014)**473.1** | 5B | 1 | [**AY4874**](https://www.ncbi.nlm.nih.gov/nucleotide/40890088?report=genbank&log$=nuclalign&blast_rank=1&RID=8BK3SUAC014)**67.1** |
| 2A | 13 | [**AY487**](https://www.ncbi.nlm.nih.gov/nucleotide/40890102?report=genbank&log$=nuclalign&blast_rank=1&RID=8BK6ZA0U014)**497.1** | 5B | 9 | [**AY487493.1**](https://www.ncbi.nlm.nih.gov/nucleotide/40890190?report=genbank&log$=nuclalign&blast_rank=1&RID=8BKK23KN014) |
| 2A | 14 | [**AY487438.1**](https://www.ncbi.nlm.nih.gov/nucleotide/40890080?report=genbank&log$=nuclalign&blast_rank=1&RID=8BJYYDZ8014) | 5B | 20 | [**AY487466.1**](https://www.ncbi.nlm.nih.gov/nucleotide/40890136?report=genbank&log$=nuclalign&blast_rank=1&RID=8BKCFTDK015) |
| 6A | 2 | [**AY487454.1**](https://www.ncbi.nlm.nih.gov/nucleotide/40890108?report=genbank&log$=nuclalign&blast_rank=1&RID=8BK8YSB1015) | 6A | 2 | [**AY487454.1**](https://www.ncbi.nlm.nih.gov/nucleotide/40890108?report=genbank&log$=nuclalign&blast_rank=1&RID=8BK8YSB1015) |
| 6A | 3 | [**AY487447.1**](https://www.ncbi.nlm.nih.gov/nucleotide/40890084?report=genbank&log$=nuclalign&blast_rank=1&RID=8BK1UNDZ015) | 6A | 3 | [**AY487447.1**](https://www.ncbi.nlm.nih.gov/nucleotide/40890084?report=genbank&log$=nuclalign&blast_rank=1&RID=8BK1UNDZ015) |

Table S3 Partial sequences for the novel nitrilases identified with corresponding clade classification

| Nitrilase I.D. | Clade | Accession  Number | Nitrilase I.D. | Clade | Accession  Number |
| --- | --- | --- | --- | --- | --- |
| *Serratia* sp. SS17 | 1A | **MG383626** | *Microbacterium* sp. SS33 | 2A | **MG383635** |
| *Rhodococcus* sp. SS4 | 1A | **MG383627** | *Microbacterium* sp. SS33 | 4A | **MG383636** |
| *Rhodococcus* sp. SS6 | 1B | **MG383628** | *Arthrobacter* sp. SS34 | 2A | **MG383637** |
| *Rhodococcus* sp. SS8 | 1B | **MG383629** | *Ochrobactrum* sp. SW2-10 | 5A | **MG383638** |
| *Erwinia* sp. SS9 | 1B | **MG383630** | *Staphylococcus* sp. SW2-31 | 2A | **MG383639** |
| *Serratia* sp. SS10 | 1B | **MG383631** | *Serratia* sp. SW2-37 | 1B | **MG383640** |
| *Serratia* sp. SS10 | 2A | **MG383632** | *Serratia* sp. SS17 | 1A | **MG383633** |
| *Serratia* sp. SS17 | 1A | **MG383634** |  |  |  |
